# Proteomic Identification of Differentially Expressed Proteins during Alfalfa (*Medicago sativa* L.) Flower Development

**DOI:** 10.3389/fpls.2016.01502

**Published:** 2016-10-04

**Authors:** Lingling Chen, Quanzhu Chen, Yanqiao Zhu, Longyu Hou, Peisheng Mao

**Affiliations:** ^1^Beijing Key Laboratory of Grassland Science, Forage Seed Lab, China Agricultural UniversityBeijing, China; ^2^Chifeng Academy of Agricultural and Animal SciencesChifeng, China; ^3^Chengdu Municipal Development and Reform CommissionChengdu, China

**Keywords:** alfalfa, flower, proteomics, pollination, senescence, MAPK, PCD

## Abstract

Flower development, pollination, and fertilization are important stages in the sexual reproduction process of plants; they are also critical steps in the control of seed formation and development. During alfalfa (*Medicago sativa* L.) seed production, some distinct phenomena such as a low seed setting ratio, serious flower falling, and seed abortion commonly occur. However, the causes of these phenomena are complicated and largely unknown. An understanding of the mechanisms that regulate alfalfa flowering is important in order to increase seed yield. Hence, proteomic technology was used to analyze changes in protein expression during the stages of alfalfa flower development. Flower samples were collected at pre-pollination (S1), pollination (S2), and the post-pollination senescence period (S3). Twenty-four differentially expressed proteins were successfully identified, including 17 down-regulated in pollinated flowers, one up-regulated in pollinated and senesced flowers, and six up-regulated in senesced flowers. The largest proportions of the identified proteins were involved in metabolism, signal transduction, defense response, oxidation reduction, cell death, and programmed cell death (PCD). Their expression profiles demonstrated that energy metabolism, carbohydrate metabolism, and amino acid metabolism provided the nutrient foundation for pollination in alfalfa. Furthermore, there were three proteins involved in multiple metabolic pathways: dual specificity kinase splA-like protein (kinase splALs), carbonic anhydrase, and NADPH: quinone oxidoreductase-like protein. Expression patterns of these proteins indicated that MAPK cascades regulated multiple processes, such as signal transduction, stress response, and cell death. PCD also played an important role in the alfalfa flower developmental process, and regulated both pollination and flower senescence. The current study sheds some light on protein expression profiles during alfalfa flower development and contributes to the understanding of the basic molecular mechanisms during the alfalfa flowering process. These results may offer insight into potential strategies for improving seed yield, quality, and stress tolerance in alfalfa.

## Introduction

Flowering represents the transition from vegetative growth to the reproductive phase and is a crucial terminal point in a plant’s life cycle. Flower development is a complicated process that is generally divided into flower induction, flower evocation, and flower organ formation (Tan and Swain, 2006). Flower sexual organs play an important role in plant sexual reproduction as they generate seeds through the union of pollen and egg cells. Pollination is a key event in the reproductive process for the control of seed setting; it is referred to as the interaction between pollen and the stigma (Li et al., 2012). Numerous genes, proteins, and environmental factors are involved in the interaction and influence pollination. However, because of the complexity of the pollen–stigma interaction, only a few flowers and ovules develop into fruits or seeds (Arathi et al., 1999). Proteomics have made great strides in understanding the pollen–stigma interaction, and focusing on the research of distinct proteins during the pollen–stigma interaction. Previous studies have mainly focused on the protein expression profile of pollen grains or tubes at different times during pistil development in several species, such as soybean [*Glycine max* (Linn.) Merr.] (Li et al., 2012), rice (*Oryza sativa* L.) (Li et al., 2016), corn (*Zea mays* L.) (Liu et al., 2010) and *Liriodendron chinense* Hemsl. (Li et al., 2014). However, very little research has focused on protein changes during flower pollination and post-pollination senescence.

Pollination triggers a series of developmental events that contributed to flower senescence in higher plants, for example, flower pigmentation changes, fading and curling of petal edges, petal senescence, etc. Furthermore, petal senescence is a visible symptom, and occurs soon after pollination (Samach and Smith, 2013). Petal senescence caused the flower dehydration, an increase in film permeability exosmosis, extravasation of micromolecular substances, and finally led to cell death and petal withering (van Doorn and Woltering, 2008). Pollination is initiated by signal transduction, and is regulated through ethylene synthesis to initiate the physiological process of petal senescence (Orzaez et al., 1999). Pollination can result in various physiological reactions, such as destructions and death of some of the cells in the pistils. When pollen grains germinate and the pollen tube grows down the pistil, PCD takes place in the conducting tissues surrounding the pollen tubes (Serrano et al., 2015).

The success of seed setting depends on successful pollination; however, during the flower-to-fruit transition, flower falling, and flower abortion always exists and only few flowers successfully develop seeds (Ruan et al., 2012). Lebon et al. (2008) indicate that seed setting is sustained by nutrients and photoassimilates exported from photosynthetically active leaves through the photosynthesis and the phloem in the inflorescence itself. Ruan et al. (2012) suggest that sugar and hormone signaling regulate seed setting, and glucose acts as a signal molecule to repress the expression of PCD genes and to promote cell division and seed set. However, there is, as yet, little understanding of the mechanism regulating early development during seed set. Post-pollination senescence is a complex multi-step process that determines the success of seed formation. The reduction in protein content is viewed as an important symbol of senescence (van Doorn and Woltering, 2008; Bai et al., 2010). Therefore, it was necessary to identify the specific protein and explore its mechanism during flower pollination and post-pollination senescence, as this would be beneficial in the understanding of successful seed setting.

Alfalfa (*Medicago sativa* L., 2*n* = 4*x* = 32) is an important forage in world-wide. It is a typical cross-pollination plant and has the characteristic of self-incompatibility. Alfalfa in the field always exhibits a lower setting percentage and a serious drop flower phenomenon with the actual seed yield at only about 4% of the theoretical seed yield (Wang Z.F. et al., 2007). Pollination and genetic variation are the main causes of this phenomenon (Martiniello, 1998; Sengul, 2006). Alfalfa has a unique flowering mechanism and a complicated pollination process that depends on external mechanical strength and the honey bee (Zhang et al., 2005). In recent years, great progress has been made in understanding the effects of pollinating insects (Shebl et al., 2009; Riday et al., 2015), tripping mechanisms (Wu and Wei, 2013), and pollination (Mol et al., 2011; Wang et al., 2011) in improving alfalfa seed set percentage. Development of the alfalfa flower is controlled by numerous genes that activate the expression of specific genes and thus the synthesis of specific proteins. Proteomics offer an effective approach to discover the proteins and pathways that are crucial for exploring flower pollination and the mechanism of fertilization at a deeper level. Such findings may be conducive to a deeper understanding of the molecular mechanism of the reproductive development of alfalfa. The current study conducted proteomic analysis on alfalfa flowers during pre-pollination, pollination, and post-pollination senescence. The aim of this study was to determine the differentially expressed proteins that are related to pollination and senescence, and to explore the possible roles of differential proteins in early development during the alfalfa seed set phase.

## Materials and Methods

### Plant Material

Alfalfa (cv. Aohan) was grown in an experimental field of the Chifeng Academy of Agricultural and Animal Sciences (longitude 118.51°E; latitude 42.17°N) in 2014. Flowers with an opening on top and all flowering at the same time were selected as samples during the full flowering stage. Artificial pollination was performed in different flowers from the same inflorescence at noon (12:00 h). Flower development was divided into three distinct stages: pre-pollinated stage (S1), fully opened with the keels still closed; pollinated stage (S2), 2 h after pollination; and post-pollination senesced stage (S3), with withering petals and shallow color (24 h after pollination). All the samples were collected and immediately frozen in liquid nitrogen, and stored at -80°C for protein extraction and qRT-PCR assays. All treatments (S1, S2, and S3) were repeated in triplicate.

### Protein Extraction

Total proteins was extracted from developing flowers according to Wang X.C. et al. (2007) in three biological replicates. Flower samples (2 g) were homogenized in liquid nitrogen. The powdered flowers were suspended in 3 mL of cold extraction buffer [50 mM Tris-HCl (pH 8.5), 5 mM EDTA, 100 mM KCL, 2% (w/v) β-mercaptoethanol, and 31% (w/v) sucrose], and further ground for 30 min on ice. Subsequently, an equal volume of Tris-buffered phenol was added and centrifuged at 6000 ×*g* at 4°C for 10 min. In the next step, 1 mL of supernatant was transferred to a 10 mL microcentrifuge tube and precipitated with four volumes of cooled precipitation solution (methanol containing 0.1 M ammonium) at -20°C overnight; it was then collected by centrifugation at 6000 ×*g* for 10 min. The supernatants were discarded and the pellets were washed three times in cooled precipitation solution and 80% (w/v) acetone containing 0.07% (w/v) *β*-mercaptoethanol, respectively. Finally, the pellets were air-dried and resuspended in solubilization buffer [7 M Urea, 2 M Thiourea, 40 mMTris, 4% (w/v) CHAPS, 1 mM EDTA, and 1% (w/v) DTT] at 25°C for 1 h. The suspension was centrifuged at 6000 ×*g* for 5 min at 4°C to remove insoluble materials. Protein concentrations were determined by the Bradford method (Li et al., 2013) using bovine serum albumin (BSA) as the standard. The quantified protein samples were stored at -20°C until further use.

### Two-Dimensional Electrophoresis (2-DE)

Proteins were initially separated using IEF. For preparative IEF, a ReadyStrip^TM^ IPG strip (17 cm, pH 3–10, non-linear; BIO-RAD, USA) were passively rehydrated overnight with 350 μL of rehydration buffer [7 M Urea, 2% (w/v) CHAPS, 2% (w/v) DTT, 0.2% v/v IPG buffers (pH 3–10, non-linear; GE Healthcare, Uppsala, Sweden), and 0.001% bromophenol blue] containing 1 mg of protein. IEF was performed for 22.5 h at 20°C using a PROTEAN i12 IEF Cell (BIO-RAD, USA) for a total run of 100 kVh (50 V for 6.5 h, 200 V for 1 h, 500 V for 1 h, 1 kV for 1 h, 5 kV for 2 h, 10 kV for 5 h and subsequently run at 10 kV until the final volt-hours reached 100 kVh). After IEF, the IPG strips were equilibrated for 15 min in equilibration buffer [1.5 M Tris-HCl (pH 8.8), 6 M urea, 30% (v/v) glycerol, 2% (w/v) SDS, and 0.002% bromophenol blue] containing 1% (w/v) DTT, followed by 15 min in equilibration buffer containing 2.5% (w/v) iodoacetamide. For second dimensional electrophoresis, gels were transferred to 13% w/v vertical SDS-PAGE gels (30% acrylamide/bis, 0.06% TEMED, 10% SDS, 10% ammonium persulfate, and 1.5M Tris-HCL pH 8.8) in PROTEAN II xi Cell (BIO-RAD, USA) until the dye line reached the end of the gel. After electrophoresis, gels were stained overnight with Coomassie Brilliant Blue (PhastGel Blue R-350, GE Healthcare) and scanned at 300 dpi resolution using an image scanner (Bio-5000 plus MICROTEK, China). Image analysis was carried out with PDQuest software (version 8.0.1, BIO-RAD, USA). Each sample was analyzed for three biological replicates. To investigate the flower protein profiles, a comparison was made between pollinated and pre-pollinated flowers. Subsequently, a second comparison was performed of the profiles of senesced flowers, pre-pollinated, and pollinated flowers. Only spots with a significant difference (*p* < 0.05) were considered as varying spots, and the protein spots with an abundance ratio of at least a fourfold change among different flower samples were selected as differentially expressed proteins and then identified by MS.

### Protein Identification by NanoLC-MS/MS

The identified protein spots were cut from 2D gels and digested with 50 mM ammonium bicarbonate in 50% acetonitrile, then dehydrated in 100% acetonitrile and dried in a Speed-Vac. The gel pieces were rehydrated with digestion solution (10 mg/ml trypsin in 50 mM ammonium) for overnight at 37°C. The resulting peptides were extracted with 30 μL 30% acetonitrile and 0.1% formic acid and shaken for 30 min. This was repeated with 10 μL 60% acetonitrile and 0.1% formic acid, and subsequently the peptides were identified using nanoLC-MS/MS. The nanoLC separation was achieved with a Waters (Milford, MA, USA) nanoAcquity nano HPLC. Nanospray ESI-MS was then performed using a Thermo Q-Exactive high resolution mass spectrometer (Thermo Scientific, Waltham, MA, USA). Raw data from the mass spectrometer were preprocessed with Mascot Distiller 2.5 for peak picking. The resulting peak lists were searched against the *Medicago truncatula* EST database of the NCBInr protein database using the Mascot 2.5 search engine. The following parameters were used in the search: trypsin enzyme specificity with a maximum of two missed cleavage, carbamidomethylation of cysteine (fixed modifications), methionine oxidation (variable modifications), peptide mass tolerance (10 ppm), and fragment mass tolerance (0.02 Da). Among the positive matches, protein identifications based on at least two independent peptides with an individual ion score > 26 or only one peptide having a ions score > 45 was accepted, and the coverage of the protein by the matching peptides should be higher than 5%, and the expect threshold *p*-value was less than 0.05 for the Mascot search. All information of matched peptides is given in Supplementary Table 1. Theoretical molecular mass (Mr) and isoelectric point (*p*I) of identified proteins were predicted by ExPASy server^1^ to check the Mr and pI of identified proteins.

### qRT-PCR Analysis

For each flower developmental stage, three replicate samples were randomly chosen and total RNA was extracted from developing flowers using TRNzol Reagent (TIANGEN Biotech, Beijing, China) according to the manufacturer’s instructions. First-strand cDNA was synthesized with specific primers (Supplementary Table 2) using PrimeScript^TM^ RT reagent Kit with gDNA Eraser (TaKaRa, Dalian, China) according to the manufacturer’s instructions. qRT-PCR was conducted on an ABI Prism 7500 Detection System (Applied Biosystems) using SYBR Premix Taq Kit (TaKaRa, Dalian, China) according to the manufacturer’s instructions. A volume of 20 μL of real time PCR reaction mixture contained 2 μL of first-strand cDNAs, 0.5 μL of 10 μM gene-specific primers (F, R), 10 μL of 2 × SYBR Green Master Mix I, and 7 μL of ddH_2_O. PCR reaction conditions were as follows: 95°C for 30 s followed by 45 cycles at 95°C for 5 s, and at 60°C for 40 s. The β-actin gene was used as a reference gene. The relative quantification (2^-ΔΔ^*^CT^*) of gene expression was evaluated using the comparative cycle threshold method.

### Functional Annotation and Classification

To determine the functional classification and biological properties of identified proteins, the identified protein sequences were mapped with GO Terms. For this, a homology search was performed for all the identified sequences with a localized NCBI BLAST searched against the NCBInr *Medicago truncatula* database. GO annotation was performed using BLAST2GO^2^ (Conesa and Gotz, 2008). The identified proteins were classified into three functional categories: CC, BP, and MF. In addition, all mapped sequences were annotated to the KEGG database^3^ accessions to obtain protein domain information. In order to obtain reliable information, we used a hypergeometric test to perform GO enrichment and pathway enrichment. *P*-values of every GO term and KEGG pathway were calculated using the hypergeometric test and those where *P* < 0.05 were denoted as having significant enrichment.

### Hierarchical Cluster Analysis of Protein Abundance

Protein abundance values were estimated using PDQuest software (version 8.0.1, BIO-RAD, USA). Each sample was analyzed for three biological replicates. Hierarchical cluster analysis was implemented in R (version 3.2.2) after normalization of the expression abundance values.

### Statistical Analysis

All experiments were replicated in triplicate. Spot intensities, GO functional enrichment, KEGG pathway, and relative quantity of expressed genes were all analyzed statistically using analysis of variance (ANOVA) by SPSS 21.0. Treatment means were separated using Duncan’s multiple range test taking *p* < 0.05 as significant.

## Results

### Protein Profiles of Alfalfa Flower at Different Developing Stages

**FIGURE 1:**
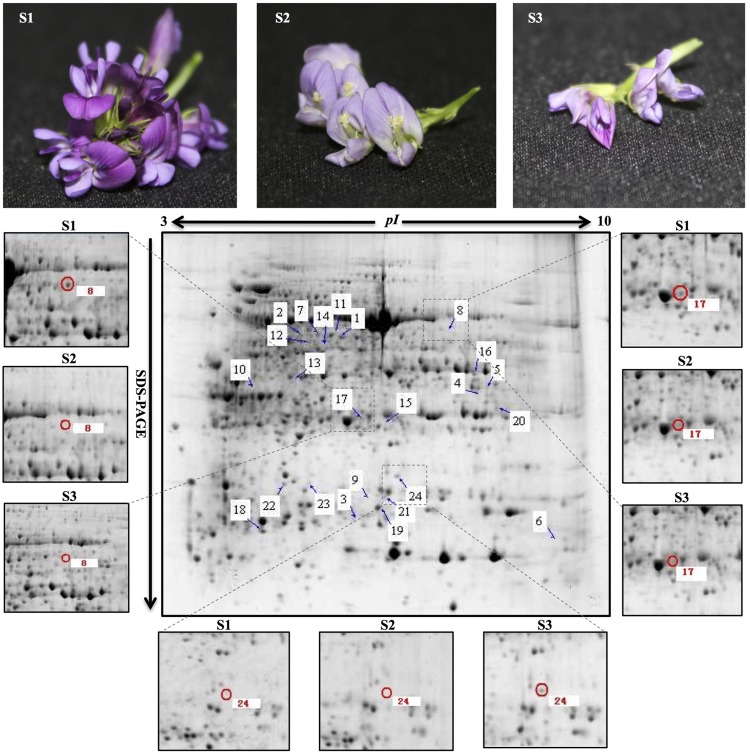
**Representative 2-DE pattern of total protein fraction from developing alfalfa flowers.** S1: pre-pollinated stage (fully opened with the keels still closed); S2: pollinated stage (2 h after pollination); S3: post-pollination senesced stage (24 h after pollination). Arrows indicate protein spots that were increased or decreased in abundance. Zoomed images show the main differentially expressed protein spots in developing alfalfa flowers. Spot 8 and 17: down-regulation in S2 and S3; spot 24: up-regulation in S3. The three biological replicates of the 2-DE gels are shown in Supplementary Figure 1.

**FIGURE 2:**
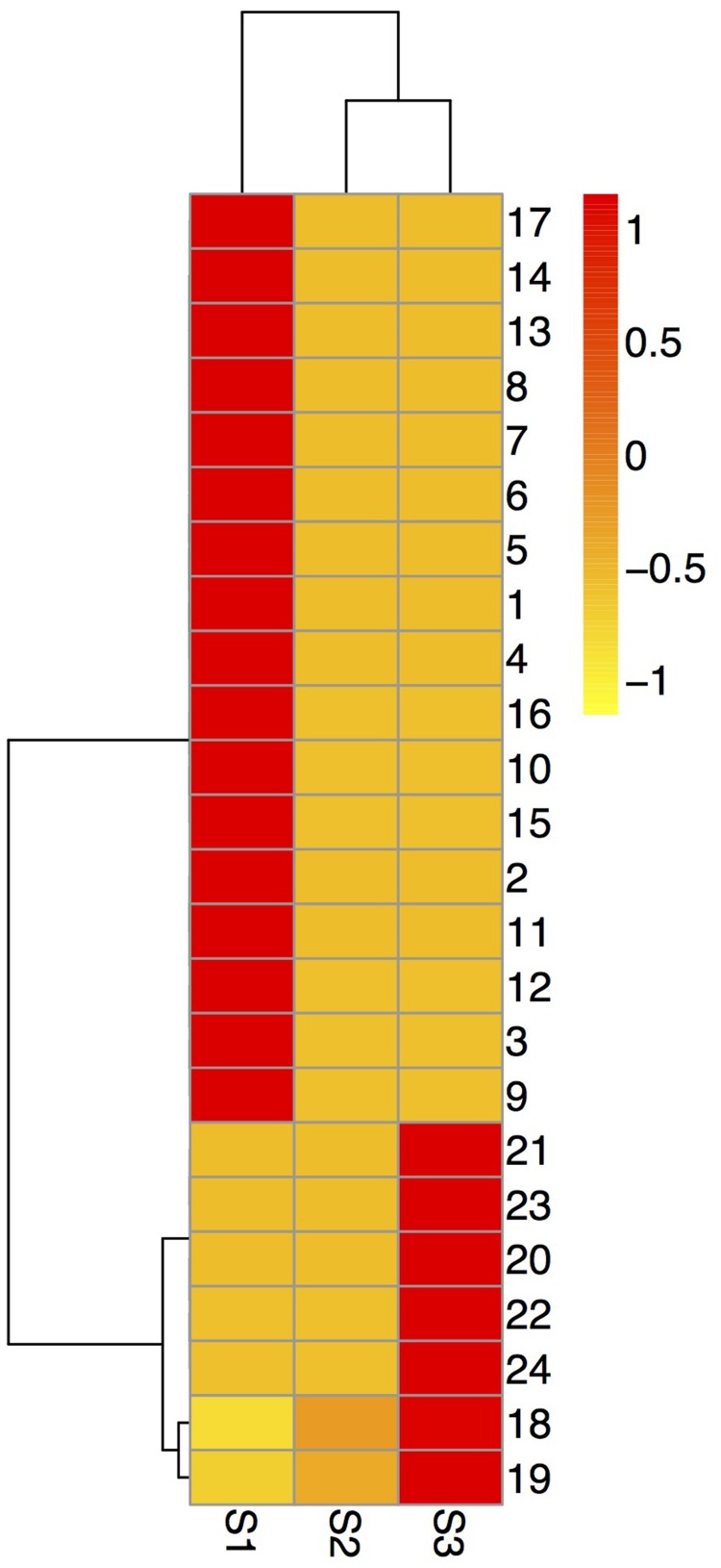
**Hierarchical cluster analysis of the expressed proteins during alfalfa flower development.** Dataset clustering was implemented in R (version 3.2.2) after normalization of the expression abundance values. Each colorized cell represents the averaged spot quantity, according to the color scale on the right of the figure. S1: pre-pollinated stage (fully opened with the keels still closed); S2: pollinated stage (2 h after pollination); S3: post-pollination senesced stage (24 h after pollination); 1-24: spot number.

According to the 2-DE with the IPG strips (pH 3–10, non-linear; **Figure 1**), the abundances of matched spots in the three replicate images were normalized and transformed using PDQuest image software. A comparison of 2-DE images revealed that there were 25 spots differed by more than fourfold (or below fourfold) in abundance between pre-pollinated, pollinated and senesced flowers. Finally, 24 spots were successfully identified by LC-MS/MS mass spectroscopy identification using a search against the Mascot software and Uniprot database retrieval (**Table 1**). The identified proteins could be divided into two types (**Figure 2**): pre-pollination expressions and post-pollination expressions proteins. Compared with un-pollinated flowers, 17 proteins (spots 1–17) were down-regulated after pollination, and seven proteins (spots 18–24) were up-regulated after pollination; most of them (spots 19–24) were expressed in senescing flowers. One spot (spot 12) did not produce a positive identification and two spots belonged to the same protein (spots 1 and 7). Furthermore, the experimental Mr of all identified proteins ranged from 13.61 to 48.81 KDa, the theoretical Mr range was from 13.66 to 48.02 KDa, and the experimental and theoretical *p*I of all identified proteins were both within the range of 4.95 to 9.47. Most of them fall within the ranges of *p*I 5.0–6.5 and Mr 16–48 kDa (**Table 1**).

**Table 1:** Differentially expressed proteins identified by LC-MS/MS during alfalfa flower development.

Spot	Protein ID	Protein name	Accession no.^a^	Score^b^	Coverage (%)^c^	Theoretical Mr/pI^d^	Experimental Mr/pI^e^
1	A0A072V8Q4	*S*-adenosylmethionine synthase	gi| 922390587	7797	79	43.60/5.77	43.17/5.77
2	G7IDU4	Protein disulfide isomerase-like protein	gi| 922396181	747	42	40.74/5.38	40.43/5.38
3	G7IFU0	Cofilin/actin-depolymerizing factor-like protein	gi| 357448329	1319	65	16.24/6.16	16.08/6.16
4	G7L2L3	NADH-cytochrome b5 reductase	gi| 357508929	79	17	31.01/8.30	30.86/8.30
5	Q2HRU6	Xyloglucan endotransglucosylase/hydrolase	gi| 357508519	90	8	34.47/8.42	34.15/8.42
6	G7IF49	Ribosomal L22e family protein	gi| 357442479	158	48	13.66/9.47	13.61/9.47
7	G7KUJ1	*S*-adenosylmethionine synthase	gi| 357511493	6386	77	43.71/5.59	43.28/5.59
8	A0A072UY58	Dual specificity kinase splA-like protein	gi| 922369199	328	29	47.52/7.80	47.38/7.80
9	A0A072UB69	Mediator of RNA polymerase II transcription subunit 7	gi| 922353693	161	17	19.43/6.08	19.38/6.08
10	B7FHQ2	Alpha-soluble NSF attachment protein	gi| 357461465	63	17	32.99/5.03	32.61/5.03
11	G8A1N5	Chromosome condensation regulator RCC1 repeat protein	gi| 357464765	1705	55	48.02/5.68	47.39/5.68
12	A0A072UTH6	Uncharacterized protein	gi| 922377082	278	22	41.15/5.46	48.81/5.15
13	A0A072TF84	Annexin	gi| 922325593	177	23	35.72/5.44	44.18/5.13
14	G7IT87	Phosphoglycerate kinase	gi| 357451633	3666	66	42.65/5.61	42.62/5.61
15	I3S2M3	GTP-binding nuclear Ran-like protein	gi| 922383336	80	23	25.54/6.38	25.22/6.38
16	A0A072VSG1	RNA polymerase II-associated-like protein	gi| 922399794	224	33	37.45/8.58	37.18/8.58
17	G7KIR1	Carbonic anhydrase	gi| 357495991	392	34	28.45/6.10	28.19/6.10
18	G7INB7	ABA-responsive protein	gi| 357449145	717	52	16.60/4.95	16.61/4.95
19	A0A072VEB8	Cytosolic class II small heat-shock protein	gi| 922397253	69	9	17.91/6.31	17.87/6.31
20	G7JC24	Enoyl-CoA hydratase/delta3,5-delta2,4-dienoyl-CoA isomerase	gi| 922375826	325	33	29.69/5.34	29.42/8.56
21	G7I9Z6	Calcium-dependent lipid-binding (CaLB domain) family protein	gi| 357438759	240	33	19.85/6.59	19.46/6.59
22	G7I8G3	Cation transporter ChaC	gi| 357438635	65	14	20.99/5.34	20.89/5.34
23	G7L7T5	Pathogenesis-related protein bet V I family protein	gi| 357515823	156	31	18.18/5.50	18.02/5.50
24	B7FMX0	NADPH:quinone oxidoreductase-like protein	gi| 357515109	45	7	21.14/6.52	21.15/6.52

*^a^Accession number from the NCBI database. ^b^MASCOT protein score from the LC-MS/MS analysis. ^c^Sequence coverage percentage. ^d^Theoretical molecular weight/isoelectric point. ^e^Experimental molecular weight/isoelectric point.*

### Functional Annotation and Classification of Identified Proteins

**FIGURE 3:**
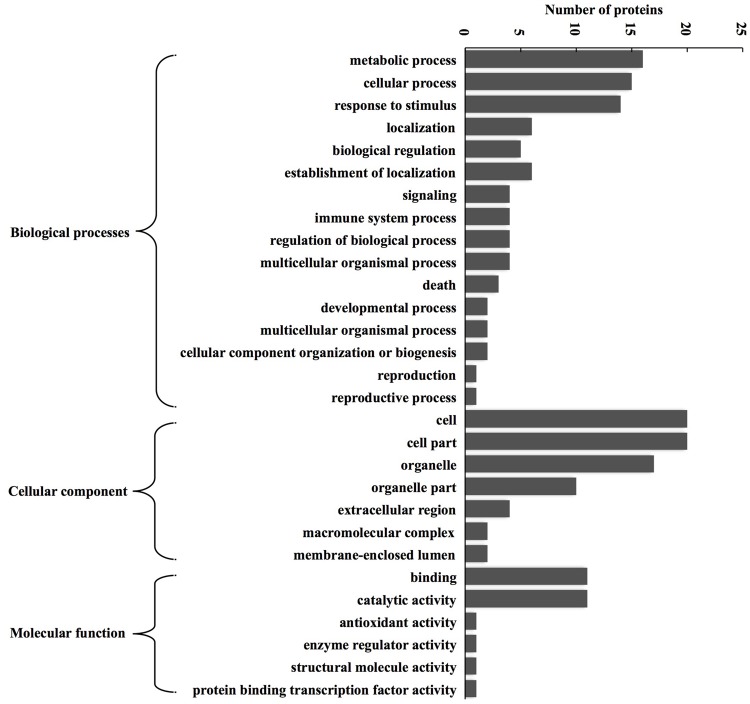
**GO classification of the identified proteins during alfalfa flower development.** Results are summarized under three main GO categories: biological process, cellular component, and molecular function. The detailed functional classification of all identified proteins is shown in Supplementary Table 3.

**FIGURE 4:**
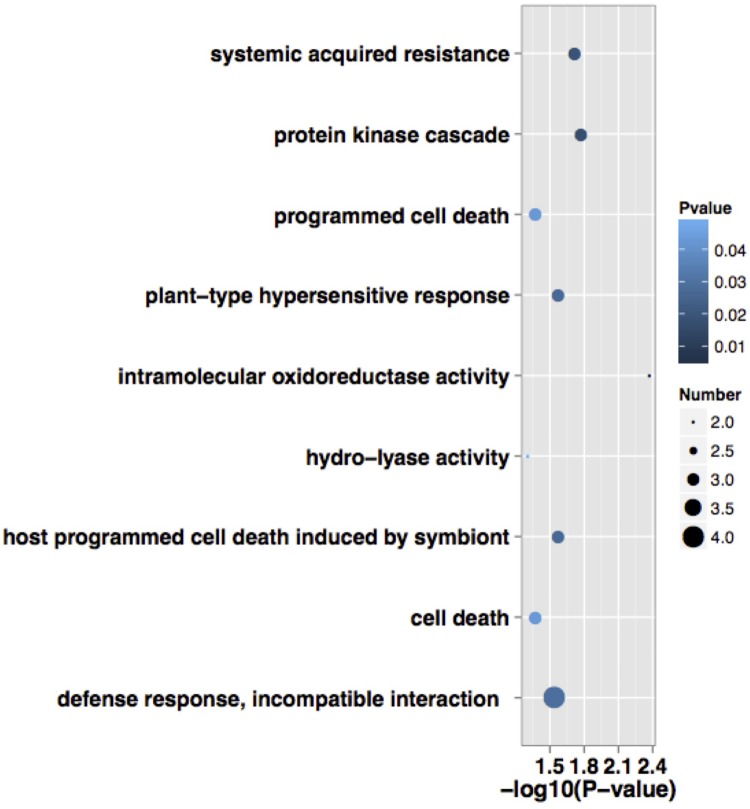
**Functional significant enrichment of the identified proteins during alfalfa flower development.** Point size indicate the numbers of annotated differential proteins, color depth indicate the *P*-value of enrichment. The detailed significant enrichment of all identified proteins are shown in Supplementary Table 4.

According to BP, CC, and MF, 24 expressed proteins were divided into 29 GO terms (**Figure 3**; Supplementary Table 3). The major functional categories in the BP were metabolic processes (16 proteins, 76.19%), cellular processes (15 proteins, 71.43%), and response to stimulus (14 proteins, 66.67%). For CC, cell (20 proteins, 100%), cell part (20 proteins, 100%), and organelle (17 proteins, 85%) were the most abundant groups, whereas binding (11 proteins, 64.71%) and catalytic activity (11 proteins, 64.71%) accounted for the most abundant groups in terms of MF. GO enrichment was further performed to elucidate the biological functions of proteins identified in alfalfa developing flowers. The results showed that there were only five proteins significantly enriched in BP and CC (**Figure 4**). Protein kinase cascade (spots 8,17, and 24), systemic acquired resistance (spots 8, 17, and 24), plant-type hypersensitive response (spots 8, 17, and 24), host PCD induced by symbiont (spots 8, 17, and 24), cell death (spots 8, 17, and 24), PCD (spots 8, 17, and 24), defense response, and incompatible interaction (spots 2, 8, 17, and 24) were significantly enriched in the BP category. Furthermore, intramolecular oxidoreductase activity (spots 2 and 20) and hydro-lyase activity (spots 8, 17, and 24) were enriched in the CC category. This result indicated that the main functions of identified proteins were signal transmission, defense response, oxidation reaction, and cell death during alfalfa flower development. Detailed enrichment information is presented in Supplementary Table 4.

In addition, KEGG analysis was used to understand the biochemical pathways of differentially expressed proteins. A total of 13 proteins were assigned to 28 pathways (Supplementary Table 5). Among these pathways, selenoamino acid metabolism was significantly enriched according to functional enrichment analysis; the related protein was SAMS (spots 1 and 7). According to standard types of KEGG pathway map, identified proteins mainly mapped as six types (metabolism, genetic information processing, environmental information processing, cellular processes, organismal systems, and human diseases) and 15 sub-types (Supplementary Table 5). Furthermore, four proteins (spots 1, 4, 7, and 14) participated in global and overview maps, carbohydrate metabolism, energy metabolism, amino acid metabolism, and metabolism of other amino acids. Four proteins (spots 2, 6, 15, and 19) participated in translation, folding, sorting, and degradation. One protein (spot 8) participated in signal transduction; two proteins (spots 3 and 20) participated in transport and catabolism, and cell motility; two proteins (spots 3 and 8) participated in the immune system, development, and cell communication; and two proteins (spots 8 and 16) participated in neurodegenerative diseases and infectious diseases.

### Transcriptional Expression Analysis of Selected Genes by qRT-PCR

**FIGURE 5:**
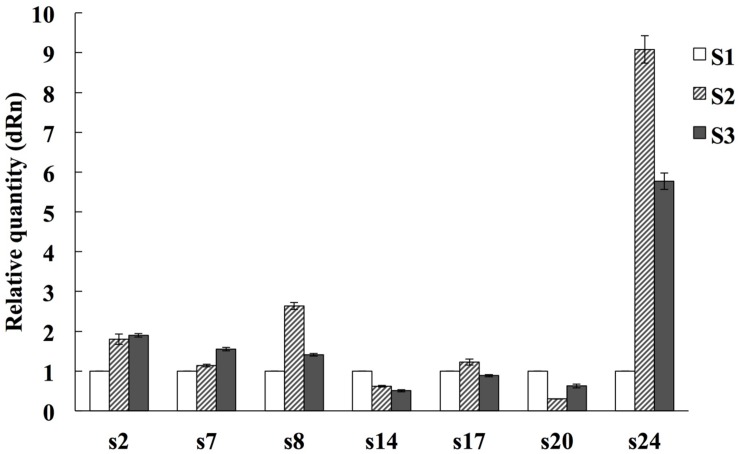
**Quantitative Real time PCR analysis on mRNA transcription of the selected expressed proteins during alfalfa flower development.** S1: pre-pollinated stage (fully opened with the keels still closed); S2: pollinated stage (2 h after pollination); S3: post-pollination senesced stage (24 h after pollination). The average expression of each gene was calculated relatively to the reference gene β-actin. This experiment was conducted three biological replicates. The relative expression value of each gene was normalized to an endogenous control and calculated using the 2^-ΔΔ^*^CT^* methods. All primer sequences are listed in Supplementary Table 2.

To confirm the proteomic results, qRT-PCR was used to examine the seven randomly selected proteins (spots 2, 7, 8, 14, 17, 20, and 24) at mRNA level using specific primers (Supplementary Table 2). Compared with the S1 stage, spots 2, 7, 8, and 24 were up-regulated at the S2 and S3 stages, spots 14 and 20 were down-regulated at the S2 and S3 stages, spot 17 was down-regulated at S3 while up-regulated at the S2 stage. The expression profiles of two genes (spots 14 and 24) at mRNA levels were consistent with those at protein levels, four gene’s (spots 2, 3, 7, and 8) expression tendency at mRNA levels were opposite to their protein levels. The mRNA and protein expression levels of spot 17 were inconsistent at the S2 stage and consistent in the S3 stage (**Figure 5**).

## Discussion

In this experiment, there were 24 proteins with over fourfold (or below fourfold) changes in abundance that were identified and functionally classified based on GO functional annotation and KEGG metabolism pathway analysis. Identified proteins were mainly related to metabolism, signal transduction, stress response and oxidation reduction, and cell death.

### Metabolism-Related Proteins Expressed during Alfalfa Flower Development

It was found that the largest group of differentially expressed proteins was related to metabolism during alfalfa flower development (**Figure 3**; Supplementary Table 5). Most of them were involved in carbohydrate metabolism, energy metabolism, and amino acid metabolism (Supplementary Table 5), and were up-regulated before pollination and down-regulated after pollination, which indicated that the primary metabolisms was enhanced to facilitate pollination. This result was consistent with previous studies in rice (Li et al., 2016) and soybean (Li et al., 2012), which suggests that enhancement of primary metabolisms in the pistil may enhance successful pollination in plants. Some common metabolic reactions, such as glycolysis, the TCA cycle, and amino acid synthesis, exist globally in plant organs and tissues. The intensity of these metabolic reactions could directly reflect plant growth conditions (Carrari and Fernie, 2006; Fait et al., 2006). Metabolism-related proteins account for 41.67% of all identified proteins during soybean flower development (Ahsan and Komatsu, 2009), and related proteins and genes account for 31 and 22% during the flowering transition of *Agapanthus* (Zhang et al., 2013). In the current study, metabolism-related proteins accounted for 38.46% of all identified proteins during alfalfa flower development, indicating that material and energy metabolism had played a crucial role during alfalfa flower development.

Phosphoglycerate kinase (spot 14) participated in glycolysis/gluconeogenesis, CYB5R (spot 4) participated in amino sugar and nucleotide sugar metabolism, and both were up-regulated in the S1 stage, but down-regulated in the S2 and S3 stages (**Figures 1** and **2**). PGK is a major enzyme involved in glycolysis and is present in all living organisms. In the glycolysis pathway, PGK catalyzes the 1,3-bisphosphoglycerate to ADP producing 3-phosphoglycerate and ATP. In the gluconeogenesis pathway, PGK catalyzes the reverse reaction (Brice et al., 2004). A deficiency of this enzyme could cause disorder in the organism’s metabolic function. Sugars have a central regulatory function in plant metabolism and growth, and are essential for germination, blooming, aging, and the stress response. Studies have shown that high concentrations of the most abundant sugars are accumulated during the early stages of floral bud development, and down-regulated during the mature pollen stage in the male sterile hybrid pummelo, indicating that the accumulated sugars supply nutrition for pollen development (Zheng et al., 2014). Sugars only were detected during pollen tube growth in *Pinus strobus* (Fernando, 2005). Similar results were observed in this study and proteins related the high accumulation of carbohydrates metabolism were up-regulated before pollination, would supply the nutrition and energy for pollen development in alfalfa flower.

Amino acids also increased during the pollination of alfalfa flowers. Cysteine and methionine are of great nutritional importance during flower development because of the limited amounts in the vegetative tissues of many plants (Azevedo et al., 2006). In the current study, SAMS (Spots 1, 7) participated in cysteine, methionine, and selenoamino acid metabolism. SAMS, an important metabolite of living bodies, catalyzed the formation of *S*-adenosylmethionine from methionine and ATP. This is a methyl donor and allows DNA methylation, it is also a precursor of polyamines and ethylene biosynthesis (Woodson et al., 1992). Ethylene is known as a plant growth regulator and it controls various biochemical reactions. Polyamine is the secondary metabolite for improving plant resistance, regulating plant growth and development, postponing senescence, and controlling morphology and architecture. It had been suggested that polyamine could regulate the plant flowering process and thus promote plant fertilization (Aloisi et al., 2016). In the current study, SAMS were up-regulate in the S1 stage, then down-regulated in the S2 and S3 stages (**Figures 1** and **2**). This process was consistent with previous studies in the stenospermocarpic table grape (*Vitis vinifera* L.) (Domingos et al., 2016) and *Petunia* (Bai et al., 2010), which indicated that SAMS were up-regulated before pollination and participated in amino acid metabolism; they were then down-regulated during corolla senescence.

Phosphoglycerate kinase (spot 14) and CA (spot 17) participated in energy metabolism. CA is involved in the nitrogen metabolism pathway; it was expressed in S1 and down-regulated in the S2 and S3 stages (**Figures 1** and **2**). With multiple cellular functions including nitrogen metabolism, photosynthesis, and water use efficiency, CA serves as an important photosynthesis enzyme during photosynthetic CO_2_ fixation, and enhances the efficiency of photosynthetic CO_2_ fixation by catalytic conversion of HCO_3_ to CO_2_ and facilitating CO_2_ supply to the cells (Badger and Price, 1994). PGK participates in the carbon fixation pathway in photosynthetic organisms. These two proteins were all related to photosynthesis. These results indicated that during the flowering process, adequate carbon sources and energy for alfalfa flower development were provided by photosynthesis. This has been confirmed in two other species, *Malus domestica* (Zeng et al., 2010) and *Brassica napus* (Sheoran and Sawhney, 2010).

### MAPK Signaling Regulates Flower Pollination and Senescence

Pollination is a crucial step in the success of seed production in flowering plants (Franklin-Tong, 2002), and exhibits various forms of cell identification and cell signal transduction. Once pollen tubes penetrate the stigma, various signaling molecules serve as key regulators for pollen tube growth. This series of dynamic cell events take place during successful fertilization and flower development (Krichevsky et al., 2007). MAPK cascades are ubiquitous signaling modules in plants and are involved in every aspect of plant growth and development, including anther development, ovule development, pollen development, pollen tube guidance, fertilization, morphogenesis, gametogenesis, embryo-genesis, abscission, senescence, and seed formation (Xu and Zhang, 2015). It had been shown that MPK3/MPK6 was regulates inflorescence architecture and pollen tube guidance in *Arabidopsis*. Double MPK3 MPK6 mutant pollen exhibited normal pollen tube growth and a normal micropylar; however, the pollen tube did not enter into the micropylar to complete fertilization, indicating that MPK3/MPK6 function was specifically involved in signal recognition between pollen tubes and the micropylar (Guan et al., 2014).

In the current study, dual specificity kinase splA-like protein (kinase splALs; spot 8), CA (spot 17), and NADPH: quinone oxidoreductase-like protein (NQOLs; spot 24) were significantly enriched during protein kinase cascades according to GO enrichment analysis (**Figure 4**; Supplementary Table 4). In KEGG pathway analysis, kinase splALs (spot 8) were mapped during signal transduction of environmental information processing, which contained MAPK signal pathways and Wnt signal pathways (Supplementary Table 5). MAPK signal pathways were detected in both GO enrichment analysis and KEGG pathway analysis.

Kinase splALs is a type of PTKs; it participates in signal transduction, cell growth, cell proliferation, and cell differentiation, and is a key element of eucaryon development (Nuckolls et al., 1996). In the present research, kinase splALs were up-regulated in the S1 stage, but down-regulated in the S2 and S3 stages (**Figures 1** and **2**). They also participated in the MAPK signal transduction pathway and other signal transduction pathways. These results indicated that kinase splALs were important signaling molecules during alfalfa pollination. As a zinc-containing metalloenzyme, CA can catalyze the reversible hydration reaction of CO_2_, and has been regarded as an important photosynthetic enzyme (Atkins et al., 1972). Light signals can regulate changes in plant structure and form, such as flower initiation, leaf expansion, stem elongation, and seed germination. CA might regulate a series of light-dependent reactions in alfalfa flower. NQOLs were up-regulated in the S3 stages (**Figures 1** and **2**), and mRNA levels of NQOLs were up-regulated in the S2 and S3 stages (**Figure 5**). However, no detailed information about any special functions is available to date. Further functional identification of these proteins would disclose their potential roles in flower senescence.

### Proteins Participating in Stress Response and Oxidative Reaction

Pollination is often affected by environmental conditions, such as temperature and pathogenic attack, etc. Furthermore, senescing petals cause a series of physiological changes, such as loss of membrane permeability, an increase in ROS, up-regulation of oxidative enzymes, and a decline in activity of certain protective enzymes (Rubinstein, 2000). Previous research has argued that there may be an overlap in the gene regulation pathways of pollination and the stress response (Lan et al., 2005). Kinase splALs (spot 8), CA (spot 17), and NQOLs (spot 24) also participate in the stress response process, and were significantly enriched during SAR, plant-type hypersensitive response, defense response, and incompatible interaction (**Figure 4**; Supplementary Table 4). SAR is a type of resistance response that occurs following an earlier localized exposure to a pathogen. An early response to pathogens in plants is the rapid death of cells in the local region surrounding an infection; this acts to prevent the spread of pathogens to other plant parts. This process is called the HR, which ultimately leads to SAR, thus enabling a resistance of the secondary infection of the pathogen (Ryals et al., 1996). Activation of MAPK cascades is one of the earliest signaling events after plant recognition of pathogen/microbe-associated molecular patterns (PAMPs/MAMPs) and pathogen effectors. It is involved in multiple defense responses, including ROS generation, HR, biosynthesis of plant stress hormones, stomatal closure, defense gene activation, phytoalexin biosynthesis, cell wall strengthening, and cell death (Ren et al., 2002). Plants detect PAMPs through pattern recognition receptors (PRRs) as attacked by pathogenic bacteria, and the MAPK cascades are responsible for accepting PRRs signals and transmitting them to downstream signal elements (Meng and Zhang, 2013). Previous reports state that tomato plants (*Lycopersicon esculentum* Mill) activate *SlMAPKKK*α to regulate PCD, and respond to a pathogenic attack (Oh and Martin, 2011). The *SlMAPKKK*𝜀 gene is related with allergic reaction and could enhance the bacterial resistance in the tomato plant (Melech-Bonfil and Sessa, 2010). Kinase splALs participated in several disease-related pathways in present study.

The reduction of CA could influence the light absorption capacity of chloroplast and thus inhibit photosystem functions and cause oxidative damage. Rice leaves maintain a high photosynthetic rate under drought conditions, which may be related to a higher CA drought responses ability (Dai et al., 2000). Yu et al. (2006) analyzed the transcription characteristics of CA in rice under drought and salt stress conditions. They found that CA expression level was up-regulated with the prolonged of drought and salt stress. They showed that there was a definite relation between rice CA and response adversity. In the current study, NQOLs protein in the stress response group was up-regulated during the S3 stages (**Figures 1** and **2**). A possible explanation is that NADPH: quinone oxidoreductase could detoxify quinone and their derivatives also reduced organelle or genetic material damage caused by the transformation of quinones, and thus maintains normal physiological functions (Gaikwad et al., 2001). The expression level of NQOLs has been shown to be significantly up-regulated (4.4-fold) in tomato leaves under salt stress (Zhou et al., 2009), whereas the mRNA levels of the corresponding EST gene are increased twofold compared with untreated seedlings (Zhou et al., 2007).

Furthermore, PDILs (spot 2) and ECH1s (spot 20) participated in the redox process (**Figure 4**; Supplementary Table 4). Electron transfer during photosynthesis and respiration is often accompanied by ROS generation. Although ROS serves as the secondary messenger in many developmental processes, excessive ROS causes oxidative damage to CCs. The evolutionary strategy of plants against oxidative damages is to generate various protective enzymes (Esfandiari et al., 2007). As one member of thioredoxin, PDILs (spot 2) showed the ability to catalyze oxidation, reduction, and isomerization of disulfide bonds in proteins that possessed chaperone and calcium ion binding sites. ECH1s (spot 20) was a mitochondrial β-oxidation, that was mapped in peroxisome metabolism (Supplementary Table 5). Peroxisome is functional in anti-disease and anti-aging processes, and is a small eukaryotic organelle within a single membrane that is specialized for carrying out oxidative reactions. Senescence is a genetically regulated oxidative process that is mainly characterized by changes of activated oxygen metabolism of peroxisomes, SOD isozymes, and ascorbate-glutathione cycle of peroxisomes (del Rio et al., 1998). Changes in activated oxygen metabolism of peroxisomes are mainly reflected by a disappearance of cascade activity and overexpression of active oxygen. It has been reported that PRX, APX, SOD, ascorbate reductase, and GSH-dependent dehydroascorbate reductase participate in the scavenging of ROS, resist internal and external stresses, and reduce oxidative stress reaction in mature rice pollen (Dai et al., 2006). Proteins related with ROS have been detected during soybean pollination. It was found that APX was up-regulated while glutaredoxin and peroxiredoxin were down-regulated after pollination, indicating that APX could detoxify hydrogen peroxide’s response to high ROS levels during soybean pollination (Li et al., 2012). ROS accumulates significantly in the stigma after pollination in rice, and SOD, APX, MDHAR, GST, CAT, and GR are expressed to different levels and participate in the scavenging of ROS (Li et al., 2016). During alfalfa flower senescence, up-regulated ECH1s had an activated oxygen-mediated role. Besides, PDILs, kinase splALs, CA, and NQOLs enriched in incompatible interaction (**Figure 4**; Supplementary Table 4), supposed that the down-regulation of CA with hydrolyase activity might have hindered the hydration reaction and reduced the water content of the stigma, and subsequently inhibited the water absorption and germination of pollen grains, and caused incompatibility between pollen and stigma. However, whether these proteins participated in the stigma–pollen interaction and the related mechanisms needs to be studied further.

### PCD Regulating Both Flower Pollination and Senescence in Alfalfa

It is well known that most changes in flowers are caused by pollination, such as ovary growth, pigmentation changes, and petal senescence. Pollination-induced senescence in petals is an essential event during sexual reproduction. It is considered a synonym of PCD, because the terms of senescence and PCD both denote the processes that initiate the programmed death of individual cells (Rubinstein, 2000). Flower senescence shows many similarities to PCD, including enhancement of hydrolytic enzymes, degradation of macromolecules, and increased in respiratory activity. Hydrolyzed products such as carbohydrates, proteins, lipids, and nucleic acids are then transported to newly growing tissues (De Michele et al., 2009). Abortion of the megaspore and microspore, degeneration of pistil primordium cells, petal withering, and pollen maturation are all accomplished through PCD during plant sexual reproduction (Rogers, 2013). Studies show that all flower cells exhibit PCD between flower induction and embryo development apart from egg cells that develop into the embryo after fertilization (Greenberg, 1996). When the pollen tube enters the stigma, the epidermal cells initiate the PCD to provide nutrition for pollen tube growth (Gonzalez et al., 1996; Greenberg, 1996).

For alfalfa flowers, it was found that 17 proteins disappeared and 7 proteins were up-regulated during post-pollination senescence. Kinase splALs (spot 8), CA (spot 17), and NQOLs (spot 24) were enriched in cell death and PCD functions according to GO functional enrichment analysis (**Figure 4**; Supplementary Table 4). Kinase splALs and CA presented to down-regulated during post-pollination senescence, while NQOLs were up-regulated (**Figures 1** and **2**). The decline of CA related with photosynthesis and nitrogen metabolism (Supplementary Table 5) was beneficial for transportation and the reuse of nitrogen sources and nutrients in reproductive organs. Kinase splALs, CA, and NQOLs also participated in MAPK signal transduction and the defense response (Supplementary Table 4). In plants, MAPK pathways are involved in the regulation of growth, development, PCD, and defense responses (Colcombet and Hirt, 2008); they also regulate plant HR cell death and PCD through disrupting the redox balance (Ren et al., 2002; Saucedo-Garcia et al., 2015). In the current study, the MAPK cascade may have regulated alfalfa petal senescence through the accumulation of kinase splALs, CA, and NQOL; however, the mechanism of senescence and cell death induced by the activation of MAPK was not clear. Although many proteins are differentially expressed during petal senescence, the functions of only a few are known.

### Comparing Protein and mRNA Levels

Simultaneous monitoring of RNA and protein expressions was performed to understand the mutual regulation between proteins and transcription levels. mRNA levels represent an intermediate state of gene expression and reflect potential protein expression. Nevertheless, some previous research considers that mRNA levels are not always consistent with protein levels, and that there is a negative correlation between mRNA and protein accumulation patterns (Mochida and Shinozaki, 2011; Lan et al., 2012). Integrated expression analysis on proteins and transcription levels could describe the overall gene–gene interaction network, and provide the function of single genes, thus enabling the exploration of its biological functions. The qRT-PCR results in alfalfa showed that only 37% of identified proteins were similar in their mRNA level, and indicated that the mRNA level couldn’t completely represent protein expression. Most differentially expressed proteins were not successfully identified according to qRT-PCR, which showed that these identified proteins might be influenced by time, environment, and other factors. Although qRT-PCR results were not completely consistent with proteome results, their similarity reached 37%, which was adequate to support the expression levels of the proteome in general. Many studies report a negative correlation between mRNA level and protein level (Zhuang et al., 2013; Nakaminami et al., 2014), existing research emphasizes the combination of transcriptome and proteome (Shemesh-Mayer et al., 2015; Li et al., 2016).

Proteins identified during alfalfa flower development are mainly related to metabolism, signal transportation, stress response, and cell death. Most proteins are involved in multiple metabolic pathways, such as the MAPK signaling pathway, stress response, oxidation reaction, and PCD. The current study provides new insight into flower developmental patterns. The results indicated that flower pollination and senescence might not be dependent upon the ethylene signal transduction pathway; however, MAPK cascades were involved in the signal transduction pathway and regulated alfalfa flower development; PCD also regulated both pollination and senescence. Related proteins such as splALs, CA, and NQOLs were vital to flower development in alfalfa. Future research will combine transcriptomes, metabolomes, and morphology in an attempt to understand crosstalk between different functional pathways and complicated regulatory mechanisms.

## Author Contributions

PM designed the study and revised the manuscript. LC carried out the study and wrote the manuscript. QC and YZ conducted the experimental work. LH carried out the bioinformatics analysis. All authors discussed the results and reviewed the manuscript.

## Conflict of Interest Statement

The authors declare that the research was conducted in the absence of any commercial or financial relationships that could be construed as a potential conflict of interest.

## Footnotes

1 http://www.expasy.org/protparam

2 http://www.blast2go.com

3 http://www.Kegg.jp/kegg/pathway.html

## Abbreviations

2DE: two-dimensional electrophoresis
APX: ascorbateperoxidase
BP: biological process
CA: carbonic anhydrase
CAT: catalase
CC: cellular component
CHAPS: 3-[(3-cholamidopropyl) dimethylammonio]-1-propane sulfonate
CYB5R: NADH-cytochrome b5 reductase
DTT: dithiothreitol
ECH1s: enoyl-CoA hydratase/delta 3,5-delta 2,4-dienoyl-CoA isomerase
GO: gene ontology
GR: glutathione reductase
GST: glutathione-S-transferase
HR: hypersensitive response
IEF: isoelectric focusing
KEGG: kyoto encyclopedia of genes and genomes
kinase splALs: dual specificity kinase splA-like protein
MDHAR: monodehydroascorbate reductase
MF: molecular function
Mr: molecular mass
MS: mass spectrometry
nanoLC-MS/MS: nanoelectrospray tandem mass spectrometry
NQOLs: NADPH: quinone oxidoreductase-like protein
PCD: programmed cell death
PDILs: protein disulfide isomerase-like protein
PGK: phosphoglycerate kinase
pI: isoelectric point
PRX: peroxidase
qRT-PCR: quantitative real-time PCR
ROS: reactive oxygen species
SAMS: s-adenosylmethionine synthase
SAR: systemic acquired resistance
SDS: sodium dodecyl sulfate
SDS-PAGE: sodium dodecyl sulfate-polyacrylamide gelelectrophoresis
SOD: superoxide dismutase
UniProt: universal protein resource.
