# Supplementary material for: Proteomic Identification of Differentially Expressed Proteins during Alfalfa (Medicago sativa L.) Flower Development
Source: Front Plant Sci. 2016 Oct 4;7:1502. doi: 10.3389/fpls.2016.01502 (PMC5047909; doi:10.3389/fpls.2016.01502)
Supplement: Supplementary file 2 [file Table_2.DOCX]

**Supplementary Table 2. Primer sequences used for quantitative real-time PCR**

| **Protein** | **Gene** | **Forward primer** | **Reverse primer** | **Product** |
| --- | --- | --- | --- | --- |
| A0A072UY58 | MTR_4g082325 | TGGTGATTCAGGACTTTAGGCAT | CTTTATCTTTGAGGTACTGGTGGAG | 159 bp |
| B7FMX0 | MTR_8g039090 | CTGATAGTGTTCTTTTTGCTTCTCC | CAGGTGGCTCAAATGCTCTTAT | 242 bp |
| G7JC24 | MTR_3g085700 | GAGTCCATCACAGCCTTAGAACGA | CGAAACCAACAATAGTGGGAAGC | 205 bp |
| G7KUJ1 | MTR_7g110310 | ACCTTAACCCTTCTGGACGCTT | GGCTGCCTGCCTTACAACATAG | 179 bp |
| G7KIR1 | MTR_6g006990 | AAATATGCTGGAACTGGATCTGC | CTCAATGAAATCAGTGGAGTAGGC | 150 bp |
| G7IDU4 | MTR_1g087520 | CAAGGGTTCTGCTTCTAGATACGG | GCCTTAGCAGGGCTGATAGACT | 138 bp |
| G7IT87 | MTR_2g066130 | CATTGGACAAGAGTCAGACCATCAT | TTACTCCCTTGCCACTTACCTCG | 122 bp |
|  | Actin | TTGAAGCCGCTGAAGGGAA | ACCAATCACTGCCACCTGCTAAT | 168 bp |
