# Supplementary material for: Proteomic Identification of Differentially Expressed Proteins during Alfalfa (Medicago sativa L.) Flower Development
Source: Front Plant Sci. 2016 Oct 4;7:1502. doi: 10.3389/fpls.2016.01502 (PMC5047909; doi:10.3389/fpls.2016.01502)
Supplement: Supplementary file 4 [file Table_4.DOCX]

**Supplementary Table 4. GO enrichment analysis of identified proteins during alfalfa flower development.**

| **GO ID** | **Gene Ontology term** | **GO class** | **Protein spot** | ***P*-value** |
| --- | --- | --- | --- | --- |
| GO:0007243 | protein kinase cascade | Biological process | 8, 17, 24 | 0.016964 |
| GO:0009627 | systemic acquired resistance | Biological process | 8, 17, 24 | 0.019181 |
| GO:0009626 | plant-type hypersensitive response | Biological process | 8, 17, 24 | 0.026767 |
| GO:0034050 | host programmed cell death induced by symbiont | Biological process | 8, 17, 24 | 0.026767 |
| GO:0009814 | defense response, incompatible interaction | Biological process | 2, 8, 17, 24 | 0.029009 |
| GO:0008219 | cell death | Biological process | 8, 17, 24 | 0.042531 |
| GO:0012501 | programmed cell death | Biological process | 8, 17, 24 | 0.042531 |
| GO:0016860 | intramolecular oxidoreductase activity | Molecular function | 2, 20 | 0.004252 |
| GO:0016836 | Hydro-lyase activity | Molecular function | 17, 20 | 0.049614 |
