# Supplementary material for: Proteomic Identification of Differentially Expressed Proteins during Alfalfa (Medicago sativa L.) Flower Development
Source: Front Plant Sci. 2016 Oct 4;7:1502. doi: 10.3389/fpls.2016.01502 (PMC5047909; doi:10.3389/fpls.2016.01502)
Supplement: Supplementary file 5 [file Table_5.DOCX]

**Supplementary Table 5. KEGG pathway of identified proteins during alfalfa flower development.**

| **Pathway class** | **Pathway name** | **Protein spot** | **Protein number** |
| --- | --- | --- | --- |
| Metabolism | Global and overview maps |  |  |
|  | Metabolic pathways | 1,7,14 | 3 |
|  | Microbial metabolism in diverse environments | 14 | 1 |
|  | Biosynthesis of secondary metabolites | 1,7,14 | 3 |
|  | Carbohydrate metabolism |  |  |
|  | Glycolysis / Gluconeogenesis | 14 | 1 |
|  | Amino sugar and nucleotide sugar metabolism | 4 | 1 |
|  | Energy metabolism |  |  |
|  | Carbon fixation in photosynthetic organisms | 14 | 1 |
|  | Nitrogen metabolism | 17 | 1 |
|  | Amino acid metabolism |  |  |
|  | Cysteine and methionine metabolism | 1,7 | 2 |
|  | Metabolism of other amino acids |  |  |
|  | Selenoamino acid metabolism | 1,7 | 2 |
| Genetic information processing | Translation |  |  |
|  | RNA transport | 15 | 1 |
|  | Ribosome | 6 | 1 |
|  | Folding, sorting and degradation |  |  |
|  | Protein processing in endoplasmic reticulum | 2,19 | 2 |
| Environmental Information Processing | Signal transduction |  |  |
|  | MAPK signaling pathway | 8 | 1 |
|  | Wnt signaling pathway | 8 | 1 |
| Cellular Processes | Transport and catabolism |  |  |
|  | Peroxisome | 20 | 1 |
|  | Cell motility |  |  |
|  | Regulation of actin cytoskeleton | 3 | 1 |
| Organismal Systems | Immune system |  |  |
|  | RIG-I-like receptor signaling pathway | 8 | 1 |
|  | T cell receptor signaling pathway | 8 | 1 |
|  | Toll-like receptor signaling pathway | 8 | 1 |
|  | NOD-like receptor signaling pathway | 8 | 1 |
|  | Fc gamma R-mediated phagocytosis | 3 | 1 |
|  | Development |  |  |
|  | Axon guidance | 3 | 1 |
|  | Osteoclast differentiation | 8 | 1 |
|  | Cell communication |  |  |
|  | Tight junction | 8 | 1 |
|  | Adherens junction | 8 | 1 |
| Human Diseases | Neurodegenerative diseases |  |  |
|  | Prion diseases | 16 | 1 |
|  | Infectious diseases: Parasitic |  |  |
|  | Leishmaniasis | 8 | 1 |
|  | Toxoplasmosis | 8 | 1 |
