# Supplementary figures and images for: Proteomic Identification of Differentially Expressed Proteins during Alfalfa (Medicago sativa L.) Flower Development
Source: Front Plant Sci. 2016 Oct 4;7:1502. doi: 10.3389/fpls.2016.01502 (PMC5047909; doi:10.3389/fpls.2016.01502)

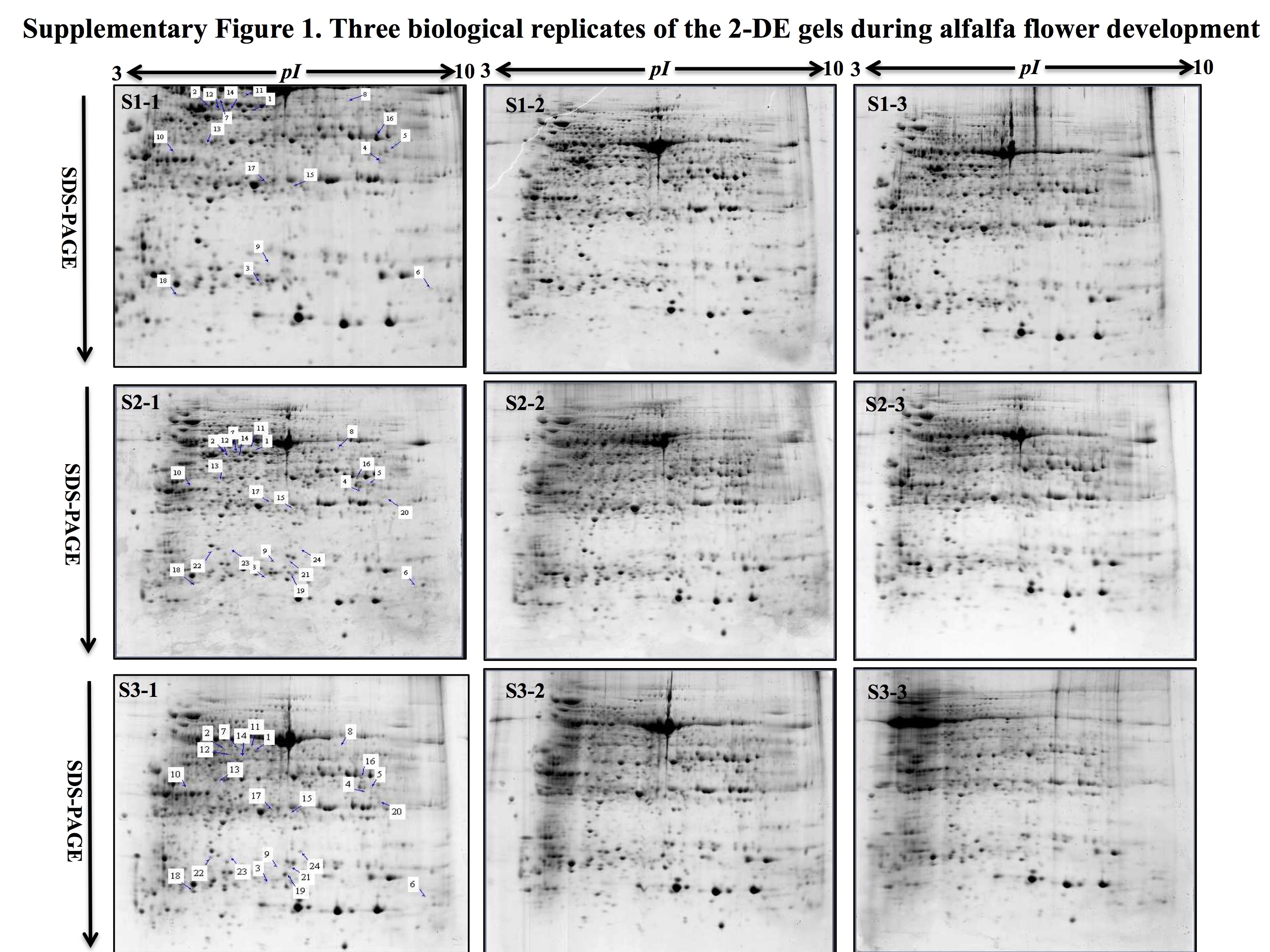

Supplement: Supplementary file 6 [file Image_1.JPEG]
